# Melatonin Pharmacological Blood Levels Increase Total Antioxidant Capacity in Critically Ill Patients

**DOI:** 10.3390/ijms18040759

**Published:** 2017-04-03

**Authors:** Giovanni Mistraletti, Rita Paroni, Michele Umbrello, Lara D’Amato, Giovanni Sabbatini, Martina Taverna, Paolo Formenti, Elena Finati, Gaia Favero, Francesca Bonomini, Rita Rezzani, Russel J. Reiter, Gaetano Iapichino

**Affiliations:** 1 Department of Pathophysiology and Transplantation, Università degli Studi di Milano, 20142 Milano, Italy; lara.damato@unimi.it (L.D.); martinatav@libero.it (M.T.); g.iapichino@unimi.it (G.I.); 2 Department of Anesthesia and Intensive Care, ASST Santi Paolo e Carlo, San Paolo University Hospital, 20142 Milano, Italy; michele.umbrello@asst-santipaolocarlo.it (M.U.); giovanni.sabbatini1@gmail.com (G.S.); formenti.paolo@faswebnet.it (P.F.); 3 Department of Health Science, Università degli Studi di Milano, 20142 Milano, Italy; rita.paroni@unimi.it (R.P.); ele.finati@gmail.com (E.F.); 4 Anatomy and Physiopathology Division, Department of Clinical and Experimental Sciences, University of Brescia, 25123 Brescia, Italy; gaia.favero@unibs.it (G.F.); francesca.bonomini@unibs.it (F.B.); rita.rezzani@unibs.it (R.R.); 5 Department of Cellular and Structural Biology, University of Texas Health Science Centre, San Antonio, TX 78229-3900, USA; reiter@uthscsa.edu

**Keywords:** melatonin, oxidative stress, critical illness, antioxidants, dietary supplements

## Abstract

In this study, the aim was to test the biochemical effects of melatonin supplementation in Intensive Care Unit (ICU) patients, since their blood levels are decreased. Sixty-four patients were enrolled in the study. From the evening of the 3rd ICU day, patients were randomized to receive oral melatonin (3 mg, group M) or placebo (group P) twice daily, at 20:00 and 24:00, until discharged. Blood was taken (at 00:00 and 14:00), on the 3rd ICU day to assess basal nocturnal melatonin values, and then during the treatment period on the 4th and 8th ICU days. Melatonin, total antioxidant capacity, and oxidative stress were evaluated in serum. Melatonin circadian rhythm before treatment was similar in the two groups, with a partial preservation of the cycle. Four hours from the 1st administration (4th ICU day, 00:00), melatonin levels increased to 2514 (982.3; 7148) pg·mL^−1^ in group M vs. 20.3 (14.7; 62.3) pg·mL^−1^ in group P (*p* < 0.001). After five treatment days (8th ICU day), melatonin absorption showed a repetitive trend in group M, while in group P nocturnal secretion (00:00) was impaired: 20 (11.5; 34.5) pg·mL^−1^ vs. 33.8 (25.0; 62.2) on the 3rd day (*p* = 0.029). Immediately from the beginning of treatment, the total antioxidant capacity was significantly higher in melatonin treated subjects at 00:00; a significant correlation was found between total antioxidant capacity and blood melatonin values (ρ = 0.328; *p* < 0.001). The proposed enteral administration protocol was adequate, even in the early phase, to enhance melatonin blood levels and to protect the patients from oxidative stress. The antioxidant effect of melatonin could play a meaningful role in the care and well-being of these patients.

## 1. Introduction

A high percentage of critically-ill patients suffer from an oxidative imbalance [1]. Guidelines suggest supplementation with vitamins, trace nutrients, and other antioxidants with the aim of restoring equilibrium [2]. Moreover, ICU patients have wake-sleep rhythm disorders [3], possibly due to the low endogenous melatonin levels [4,5].

Melatonin is an indole amine with hypnotic, antioxidant [6], and antiseptic actions [7,8], and its endogenous levels exhibit a circadian rhythm. Endogenous melatonin concentrations are decreased in ICU patients, both in terms of night time peaks and in the basal diurnal levels [9]. It remains unknown if these low levels are due to a reduced endogenous production or a result of increased metabolism [10,11]. On the one hand, mechanical ventilation and many drugs commonly used in the ICUs (e.g., benzodiazepines, steroids, β-blockers, local anaesthetics, α2 agonists, nonsteroidal anti-inflammatory drugs) inhibit melatonin production [3]. The second possibility is consistent with the redox imbalance common in critically-ill patients, especially if they are septic [9]. Since melatonin is a powerful antioxidant [12,13], its levels are typically depleted under high oxidative stress conditions. As examples, reduced melatonin levels are related to age, sepsis severity [14], post-traumatic stress disorder [15], and to the severity of sleep disruption during critical illness [10].

In situations of decreased endogenous melatonin levels (such as delayed sleep phase disorder) or during phase-shift situations (such as jet-lag), exogenous melatonin administration is effective as a circadian rhythm synchronizer [16]. In ICU patients, oral supplementation induces pharmacological levels in the short term [17,18], but this has never been tested for more than four continuous days [19]. Melatonin is a safe and inexpensive drug; it was found to have beneficial effects with regard to both sleep disruption and sepsis [20,21].

Melatonin’s therapeutic potential in ICU patients is represented by its physiological hypnogogic action (sedative saving and improved sleep quality) [11,22]. Moreover, it could be important for its antioxidant and immunomodulatory effects, as was shown in a septic shock animal model [8,23,24], in neonatal sepsis [20], in cardiovascular diseases [25], and in prostate cancer [26].

Recently, a randomized, controlled clinical trial in ICU patients [27] described the clinical effects of prolonged, enteral melatonin supplementation in reducing the need for sedation (lower requirement of neuroactive drugs), in cost reduction, and in overall clinical outcome improvement. The aim of the present study is to describe circulating melatonin levels and their relationship with the biomarkers of oxidative stress, oxidant protection, inflammation, immune response [28,29], and apoptosis [30]. This approach permitted both the control of the adequacy of enteral adsorption and a verification of the relationship between melatonin blood levels and the redox imbalance typical of critically ill patients.

## 2. Results

### 2.1. Patients Case-Mix

During the 30 months the trial was open, 82 patients participated in the exogenous melatonin randomized, controlled trial; 64 of them were also enrolled for the present part of the study at the 3rd ICU day, according to their estimated mechanical ventilation length. Among the 64 patients enrolled, 19 had a mechanical ventilation length of less than 8 days and were subsequently excluded. Some blood samples collected were excluded because there was not an adequate amount of serum from which to make the measurement. For this reason, some blood sampling series were not complete, particularly for lymphocytes.

The clinical and demographic characteristics of the studied patients are summarized in Table 1. The two groups were similar at baseline. The most frequent admission type is medical, mainly for pneumonia or lung diseases; patients had a high Simplified Acute Physiology Score (SAPS) II score at admission and presented a relatively long mechanical ventilation length, allowing them to be considered as long term ICU patients.

### 2.2. Blood Melatonin Values

**Figure 1:**
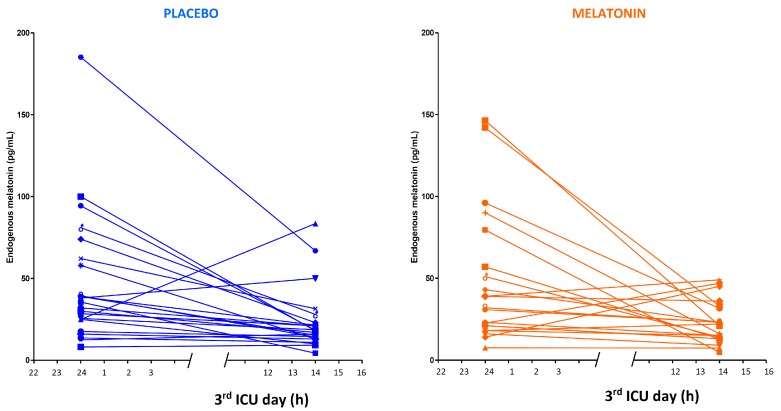
Endogenous melatonin secretion in the 3rd day of ICU measured at midnight and 14:00 h, in patients assigned to the two treatments groups before placebo or melatonin tablet administration. The median (25; 75 percentiles) values were 33.8 (25; 62) vs. 32.0 (21; 57) pg/mL at midnight and 16.8 (13; 23) vs. 21.0 (13; 32) pg/mL at 14:00 h, for the two treatment groups, respectively, without significant differences.

**Figure 2:**
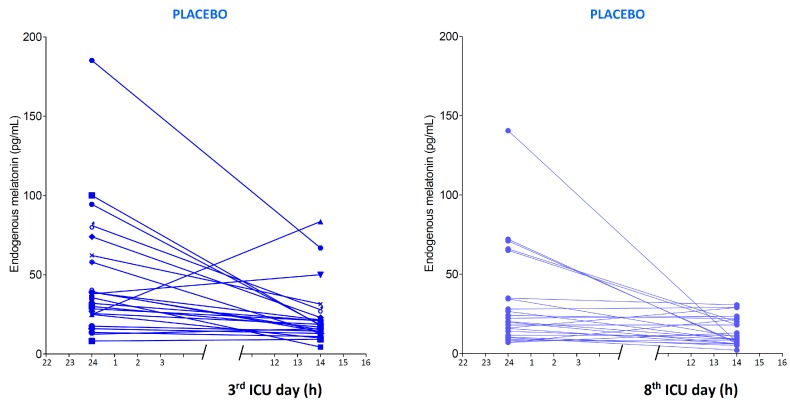
Endogenous melatonin secretion in the 3rd and 8th day of ICU measured at midnight (24) and at 14.00 h (14), in patients treated with placebo. The median (25; 75 percentiles) values were 33.8 (25; 62) vs. 20.0 (12; 35) pg/mL at midnight and 16.8 (13; 23) vs. 10.9 (7; 22) pg/mL at 14:00 h, at the 3rd and 8th day, respectively.

The endogenous melatonin secretion during the baseline observations was similar in the two randomized groups of patients. High interindividual differences were found, together with a partial preservation of the circadian rhythm (Figure 1). The medians (interquartile range) were 34 (25; 62) for placebo patients vs. 32 (21; 57) pg·mL^−1^ for the melatonin group at midnight and 17 (13; 23) vs. 21 (13; 32) pg·mL^−1^ at 14:00 h, without significant differences (Table S1). In the later phase of critical illness (8th ICU day), the nocturnal melatonin secretion in placebo patients seemed to be impaired in comparison with the 3rd ICU day: 20 (12; 35) pg·mL^−1^ at midnight (*p* = 0.029), while in the daytime there was no statistical difference: 11 (7; 22) pg·mL^−1^ (Figure 2).

**Figure 3:**
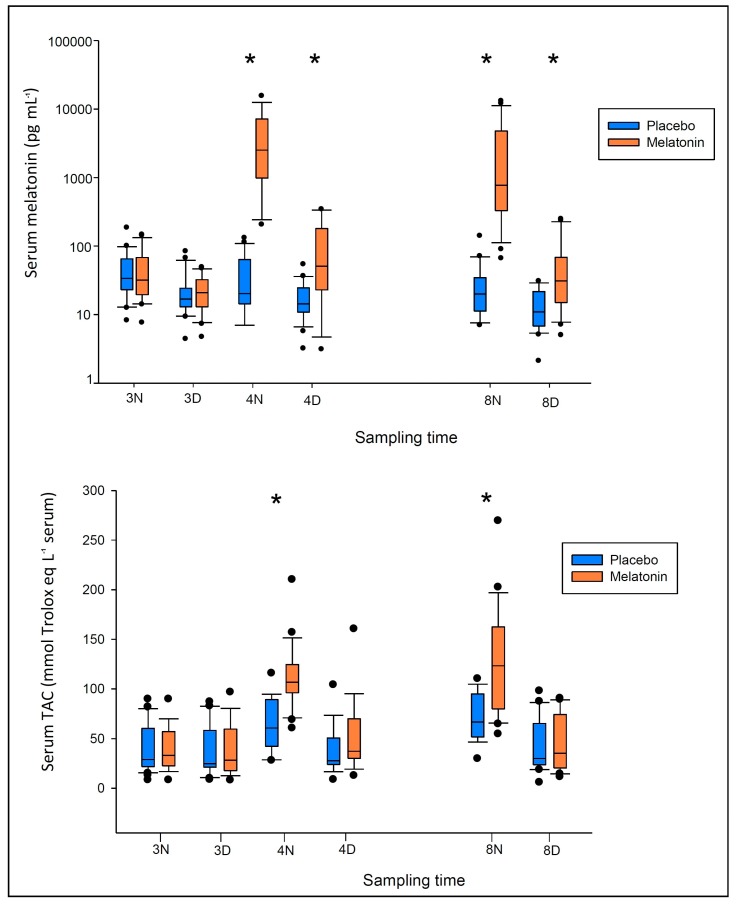
Serum melatonin and total antioxidant capacity (TAC) values measured in critically-ill patients at midnight (N) and at 14:00 h (D) of the 3rd, 4th, and 8th ICU day. Patients were randomized to receive either melatonin 3 mg at 20:00 h and midnight or placebo from the 4th ICU day. Data are represented as “box and whiskers” plots: within each plot, the box is bordered at the first (Q1) and third (Q3) quartile of the variable, and is cut by a line corresponding to the median; whiskers extend from the box to the 95% confidence interval. Dots represent outlier values. Comparisons were made by Wilcoxon rank-sum test for unmatched data. * denotes *p* < 0.05 between groups.

After exogenous enteral melatonin administration, the plasma levels of the indole rose significantly with respect to the placebo controls, even in the early treatment phase (Figure 3, Table S1). The levels reached pharmacological values in all patients [17,31] after 4 h from 3 mg tablet administration: 2514 (1106; 6353) for the melatonin group, 21 (15; 65) pg·mL^−1^ for the controls (*p* < 0.001); the differences with endogenous levels were statistically significant also after 14 h: 51 (28; 170) vs. 14 (51; 28) pg·mL^−1^ (*p* = 0.01).

This is also evidenced by the differences between the concentration/time profiles in the early phase: 53,413 vs. 1221 pg·h·mL^−1^ for the melatonin vs. placebo (*p* < 0.001) (Figure S1). In the late treatment phase, after 5 days of melatonin supplementation no accumulative effect was observed either at midnight or at 14:00 h, with circulating blood values being not different to those after the 1st administration (midnight: 2514 (982; 7148) pg·mL^−1^ at 4th ICU day vs. 775 (333; 3545) pg·mL^−1^ at 8th ICU day, *p* = 0.086; afternoon: *p* = 0.208, Table S1).

In Table S2, the blood melatonin levels are expressed as differences from the 3rd day (baseline) to 4th (early) and 8th (late) ICU days. In addition, this analysis confirms that the treatment produced a significant positive difference in blood melatonin both in the early and in the late ICU period with respect to the 3rd ICU day. By comparison, endogenous blood melatonin levels in the placebo patients showed a negative trend, with a progressive reduction in the late stages.

### 2.3. Serum Antioxidant Capacity

**Figure 4:**
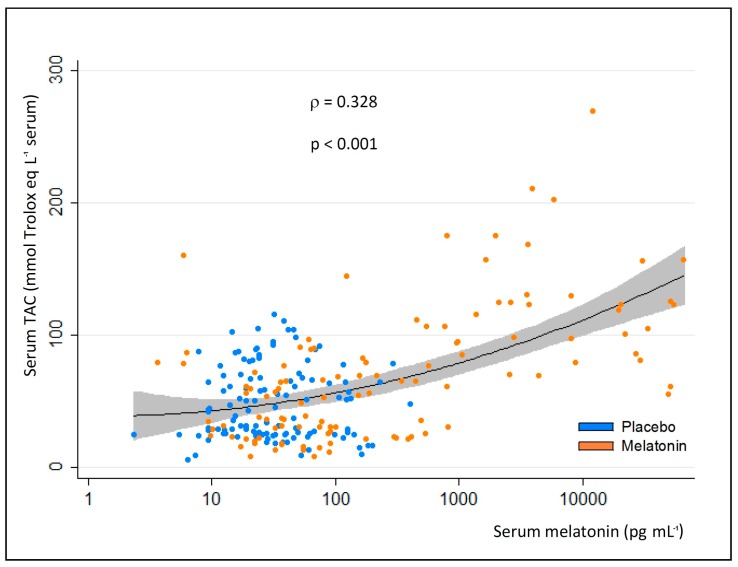
Correlation between serum melatonin and serum total antioxidant capacity (TAC) measured in all available samples, taken from critically-ill patients at midnight and at 14:00 h of the 3rd, 4th, and 8th ICU day; the patients had received melatonin or placebo. Analysis was done by Spearman rank correlation. ρ: Spearman correlation coefficient. Line represents the linear prediction; gray belt is the 95% confidence interval; points are all the available couples of observed values.

Figure 3 (lower panel) shows the behavior of serum total antioxidant capacity (TAC). After 4 h of the first melatonin administration (20:00), the serum of the melatonin patients was more protected against oxidant insults, displaying a significantly higher antioxidant capacity with respect to the placebo controls. This same behavior is replicated in the late phase. Overall, the total serum antioxidant capacity shows a significant correlation with melatonin levels (Figure 4).

By determining the differences from the 3rd and subsequent ICU days (Table S2), TAC was significantly different in melatonin patients at the 4th and 8th night, while values did not change during the day. The same trend, even if less evident, was shown in the placebo patients, with a significant increase with respect to the 3rd night during the 4th (*p* = 0.004) and 8th night (*p* < 0.001), and no differences during the daytime observations.

### 2.4. Redox Imbalance in Serum

The nighttime levels of circulating hydroperoxides did not suggest an oxidative imbalance associated to oxidant-antioxidant disequilibrium. These values were 4.5 (2.9; 6.4) for placebo vs. 4.7 (3.4; 5.7) H_2_O_2_ mmol·L^−1^ in the melatonin patients on the 3rd night, 4.4 (3.7; 5.5) vs. 3.7 (3.0; 6.3) on the 4th night, and 4.1 (3.9; 5.1) vs. 3.8 (3.2; 5.4) on the 8th night (normal range: 4.7–7.1 H_2_O_2_ mmol·L^−1^) (*p* > 0.05 for all comparisons between and within groups). In this context, neither melatonin treatment nor the time spent in the ICU, caused substantial changes in redox imbalance, with values always around the lower normal range.

### 2.5. Lymphocytes

**Figure 5:**
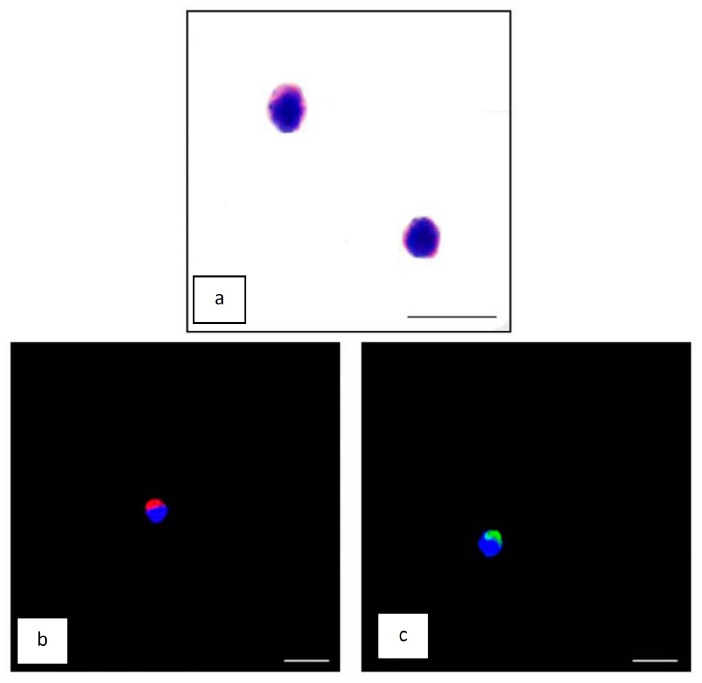
May Grünwald-Giemsa-stained lymphocyte of a control subject (**a**); and a confocal image showing inducible Nitric Oxide Synthase (iNOS) positivity (**b**); and cytochrome C positivity (**c**) lymphocytes of a placebo treated critically-ill patient. Scale bar = 20μm.

The isolated lymphocytes stained with the May Grünwald-Giemsa stain showed classical morphological features with a huge and round nucleus and a thin and basophilic cytoplasm in both groups, similar to the control subjects (Figure 5a). Furthermore, most of the lymphocytes from Ficoll-isolated peripheral blood expressed both inducible nitric oxide synthase (iNOS) and cytochrome C; particularly obvious was iNOS (red staining) and cytochrome C (green staining) immunopositivity, both observed in the cytosol (Figure 5b,c). No significant differences in iNOS or cytochrome C immunopositivity were observed between critically-ill patients treated with placebo or with melatonin (Tables S3 and S4, Figure S2).

## 3. Discussion

The results presented herein are a subset of that recently published [27], related to the clinical effects of melatonin treatment in critically-ill patients. Exogenous melatonin administration led to a decreased need for analgesics and sedative drugs, speeding the weaning from neuroactive drugs and from mechanical ventilation, and improving some neurological status indicators such as pain, anxiety, and agitation. Sleep time was shorter during the daytime and longer during the night, indicating an influence on the circadian rhythm. Moreover, melatonin-treated patients seemed to have a less severe septic state, with lower organ dysfunction, white blood cell count, total blood bilirubin, and need for vasoactive drugs. 

### 3.1. Melatonin Blood Levels

The attainment of pharmacological levels of melatonin in the blood confirm that gastro-intestinal absorption of melatonin is adequate even in the early phase of critical illnesses [10,15,16]. 

With respect to endogenous nightly secretion, the melatonin treated ICU patients presented 2-log higher melatonin levels 4 h after the administration. Even in the daytime, 14 h after the 2nd administration, the levels were still significantly higher. Repeated treatment with melatonin did not result in a further serum accumulation until the 8th ICU day. The published pharmacokinetic parameters [17,18] indicate a rapid metabolism and/or cellular uptake, roughly maintained even after 5 treatment days. This observation suggests a novel scenario for the possible development of different formulations and different dosages that may more closely mimic endogenous levels. It would be of interest to test if a prolonged release, transdermal administration, or a lower dosage may be effective as well as exhibiting the same clinical and biochemical parameters.

Regarding the observations made on the placebo-treated patients, even if nocturnal melatonin peaks were decreased, the circadian rhythm was maintained. A trend for a reduction in the nocturnal blood levels in the late ICU phase was found. The lower melatonin blood levels may mean an exhaustion in endogenous production or increased consumption, both capable of causing a disproportioned change between need and availability [27,31,32]. Overall, melatonin blood levels did not show any relationship with illness severity or excretory organ activity, but the power was not adequate to prove this.

### 3.2. Antioxidant Status

Antioxidants play an important role in preventing formation and in scavenging free radicals and other potentially toxic oxidizing species. Herein, it was observed that the serum antioxidant activity due to the sum of all antioxidant species, i.e., enzymes (GSH reductase, catalase, peroxidase, etc.), small molecular scavengers (ascorbate, uric acid, GSH, vitamin E, etc.) and proteins (albumin, transferrin, etc.), provided an indication of the overall capability to resist oxidative damage. ICU patients are at risk for oxidative damage and, even if they have regular administration of a full dose of recommended vitamin supplementation [33], they present TAC values lower than reported in the literature [34]. As observed in other diseases [35], melatonin seems to be effective in increasing the protection against the total antioxidant damage, by normalizing TAC values. The association between TAC and circulating melatonin suggests that only pharmacological levels influence antioxidant values in a significant manner. Consistent with this is that ICU patients receiving placebo showed a significant higher antioxidant status at midnight with respect to the afternoon (14:00 h), probably reflecting the higher melatonin secretion and the lower energy requirements at night [36]. 

The D-Roms test provides a measure of hydroperoxides (Reactive Oxygen Metabolites) circulating in the serum. The production of these species by oxygen free radicals may exceed the antioxidant defenses of the organism. If pro-oxidant conditions exist, polyunsaturated fats are transformed into alkoxyl (RO^●^) and peroxyl (ROO^●^) radicals, which ultimately amplify the oxidative damage in all cells. Interestingly, even if the ICU patients are at risk of oxidative damage, values of hydroperoxides < 5.9 H_2_O_2_ mmol·L^−1^ were always found during the nighttime. These values are generally indicative of the presence of adequate antioxidants or of a very high potential antioxidant protection, typical of healthy people [37]. In ICU patients, the higher nighttime antioxidant potential could be due to the special regimes including vitamin supplementation, enteral nutrition, or particular medical treatments.

The patients showed an overall normal oxidative state associated with an under normal TAC, indicating they were receiving adequate therapy, sufficient to cope with any oxidative imbalance. In this context, melatonin supplementation coupled with physiological levels, led to a trend to restore the normal TAC values.

### 3.3. Observations Made on Lymphocytes

To have a comprehensive view of the pro- and anti-oxidant status of our patients, the expression of iNOS and cytochrome C were assayed in lymphocytes. iNOS is upregulated specifically in activated cells of immunity and is involved in the inflammatory reactions and oxidative stress processes that cause cytotoxic changes. Cytochrome C is an apoptogenic protein crucial for activating the caspase cascade of cell degradation. In this study, the analyses made on lymphocytes showed no significant differences between melatonin and non-melatonin treated patients. This may be related to the fact that the cellular milieu of critically-ill patients is highly complex and heterogeneous, and because they receive a number of drugs that impact the pro-oxidant/antioxidant status. 

### 3.4. Study Limitations

A rather low number of critically-ill patients was enrolled in this study, and a high number of samples was not analysed for logistical problems as described above. Second, a very heterogeneous cohort of critically-ill patients was studied; the only common characteristic they had was the mechanical ventilation for a duration of more than 8 days. Third, only one parameter each of oxidative stress (hydroperoxides) and nitrosative stress (iNOS) was monitored, instead of a panel of biomarkers. Fourth, the present study lacks the enrollment of a healthy people group, so we can only compare the results obtained in critically-ill patients with those reported in the literature on healthy people. For the current investigation, we were interested in describing the effects of the two planned treatments in a very particular model of critically-ill patients. Fifth, since only one dose regimen of melatonin was used here (3 + 3 mg daily), a dose-effect relationship on the oxidative stress was not possible to describe.

## 4. Materials and Methods 

### 4.1. Study Design

**Figure 6:**
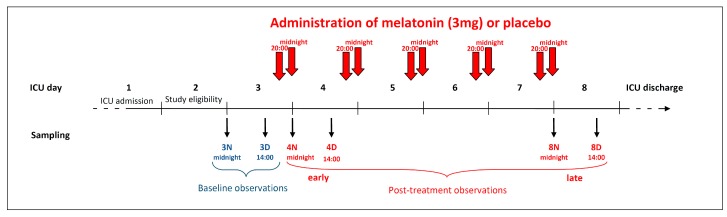
Timeline of the study. The patients were admitted to the ICU on day 1, and enrolled in the study during day 2. During the 3rd ICU night and day (midnight and 14:00 h), blood samplings were done to measure the baseline blood melatonin and total antioxidant capacity. At 20:00 of the 3rd ICU day, treatment with melatonin or placebo was begun: each patient received a 3 mg tablet of melatonin at 20:00 h and midnight (total 6 mg daily), until ICU discharge. Post-treatment blood samples were collected both in the early (4th night and day) and in the late (8th night and day) periods.

Among the 82 patients participating in the MelaSed trial (Trial Registration: Clinicaltrial.gov number: NCT00470821) [30], 64 patients with an estimated length of mechanical ventilation at ICU admission higher than 8 days were considered for enrollment in this a priori stated biochemical part of the study (male 36/64). The first two ICU days represented the pre-study period for each patient (Figure 6). To describe the endogenous melatonin levels, blood samples were drawn during the 3rd ICU day, at 00:00 and at 14:00, to roughly investigate the melatonin nocturnal peak and the subsequent daytime nadir. The patients’ beds were always oriented toward windows open to the sunlight, while during the night, extreme attention was paid to maintaining darkness. Blood samples were always taken via central venous catheters that were previously inserted.

Starting from the evening of the 3rd ICU day and continuing until ICU discharge, each patient received a 3 mg melatonin tablet at 20:00 and at 24:00 (total 6 mg melatonin per day) or placebo tablets without the active ingredient. On the 4th and 8th days, blood samples were obtained at the same time points of the 3rd day (at 00:00, i.e., 4 h after the previous melatonin administration but immediately before the next one), and at 14:00, 14 h after the previous administration (Figure 6).

Analgesics (morphine or fentanyl), sedatives (enteral hydroxyzine and lorazepam, intravenous propofol and midazolam), and antipsychotics (haloperidol) were administered based on clinical rational as were other treatments. In particular, all patients received antioxidant agents according to hospital guidelines (daily administration of vitamin C, E, and group B at midday) and insulin (continuous intravenous administration to maintain blood glucose between 80 and 150 mg·mL^−1^).

#### 4.1.1. Eligibility 

All patients admitted to the general ICU of a University Hospital (A.O. San Paolo—Polo Universitario, Milano, Italy) between July 2007 and December 2009 were screened for enrolment in the clinical part of the present study. Because of the inclusion and exclusion criteria [27], any patient that did not have collection of all 6 blood samples (from 3rd to 8th ICU day) was excluded.

#### 4.1.2. Ethics

The study was approved by the local Ethics Committee (#54/2006, 25 October 2006). Written informed consent was collected from able patients and a written declaration of received information was collected from relatives of the others, as per local Ethics Committee requirements. As soon as their neurological conditions improved, patients were duly informed of the study and their written consent was obtained both for their previously-collected data and for further randomized treatments. After informed consent, a sealed brown envelope was assigned to patients during the first 2 ICU days; it was then opened during the morning of the 3rd ICU day, randomly assigning each eligible patient to the melatonin or placebo group. No more data were gathered or were excluded from the database if patients did not confirm their consent.

#### 4.1.3. Randomization, Masking, Tablet Preparation and Administration, and Blood Measurements

Treatment allocation was obtained through a computer-generated eight-patient block randomization procedure, with the parallel assignment of patients, and a 1:1 ratio between groups. 125 mg tablets containing 3 mg of pure melatonin (Helsinn, Biasca, Switzerland), and microcrystalline cellulose (70 mg), calcium phosphate (47 mg), magnesium stearate (2.5 mg), and sodium carboxymethyl cellulose (2.5 mg) were used (Procemsa, Torino, Italy). Similar tablets without melatonin, for the patients assigned to the placebo group, were also prepared. All tablets were administered by naso-gastric/naso-jejunal tube or by ileostomy, after crushing the tablet and mixing it with 20 mL of water, followed by another 20 mL to flush out the residue. The appearance of the uncrushed and crushed melatonin and placebo tablets was identical, the two groups being indistinguishable for nurses or physicians [27]. 

### 4.2. Blood Samples Management

Blood samples were immediately processed. One sample of 2.7 mL was collected in tubes without an anticoagulant agent; after 10 min for serum sedimentation, it was centrifuged at 2452× *g* (4000 rpm with radius 13.7) for 10 min at 4 °C and the serum was stored at –80 °C until analysis. Melatonin values and oxidative status evaluations were all performed together at the end of the study. Another 5 mL blood sample was taken in a heparinised tube for lymphocyte separation. First, each sample was diluted 1:1 with Roswell Park Memorial Institute (RPMI) medium under a sterile hood. Then, it was carefully transferred over Ficoll and centrifuged at 613× *g* (2000 rpm with radius 13.7 cm) for 30 min at 4 °C excluding the brake. Thereafter, the white ring containing lymphocytes was transferred into another tube containing phosphate buffered saline solution (0.1 mol/L; pH 7.4, 1:1, *v*/*v*) and centrifuged at 300× *g* (1400 rpm with radius 13.7 cm) for 10 min at 4 °C with the brake on. After the supernatant was removed, the lymphocyte pellet was suspended in 1 mL freezing solution (50% fetal calf serum, 40% RPMI medium, 10% Dimethylsulphoxide, DMSO) and stored at −80 °C until analysis.

### 4.3. Melatonin Assay

Melatonin was assayed in serum by a competitive enzyme immunoassay (Melatonin ELISA REF RE54021; IBL, Hamburg, Germany) that includes sample pre-purification by solid-phase extraction (SPE) on C18 RP columns provided by the manufacturer. Aliquots (0.5 mL) of the samples, controls, or calibrators were purified by SPE following the manufacturer’s instructions, dried under nitrogen and stored at −20 °C for up to 48 h. Serum samples from melatonin-treated patients suspected to contain concentrations higher than the highest standard (300 pg/mL) were diluted 1:50 (*v*/*v*) with diluted assay buffer prior to the extraction step. Dried extracts were reconstituted with 0.15 mL of bi-distilled water and 0.05 mL was transferred in duplicate into the microtitre plate. After processing as described by the manufacturer’s instructions, the microplate was read at 405 nm. By considering B = OD standard, B0 = OD blank, Logit B/B0 = LN [(B/B0)/(B/B0_1)], the concentration of serum melatonin in pg/mL (i.e., ng/L) was calculated by plotting logit B/Bo on the *y*-axis versus LN of the melatonin concentration (LN pg/mL) on the *x*-axis. In the case of diluted samples, the final value was multiplied by the corresponding dilution factor. Samples showing concentrations above the highest standard were re-assayed after appropriate dilution. The sensitivity of the assay was 1.6 pg/mL. Both intra- and interassay coefficients of variation were <20%.

### 4.4. Serum Antioxidant Capacity

The serum antioxidant capacity was assayed using a colorimetric assay kit (Total Antioxidant Capacity Assay kit Catalog #K274-100; BioVision, Inc., Mountain View, CA, USA) that measures Cu^++^ reduction to Cu^+^ by the antioxidant factors in the sample by coupling with a colorimetric probe. For calibration, 1 mM Trolox in DMSO:water was used. Each microtitre plate was filled with either 0.1 mL calibrators (0, 4, 8, 16, or 20 nmol Trolox) or 0.1 mL diluted serum (1:2000 *v*/*v*, equivalent to 0.25 μL). Then, 0.1 mL of freshly prepared Cu^2+^ working solution was added into each well and the mixture was incubated at room temperature for 1.5 h. The optical density was determined at λ = 540 nm in a microplate reader. The standard Trolox (nmol/well) versus optical density curve was used to obtain the sample antioxidant capacity expressed as nmol Trolox equivalent/μL serum (i.e., mM Trolox equivalents): [(sample OD-blank OD)/(slope of standard curve in nmol)]/undiluted serum volume (μL) added to the wells.

### 4.5. Redox Imbalance of Serum

To determine the serum oxidative stress level, nocturnal blood samples were used only when melatonin levels were expected to be at the highest values, and the capacity of in vivo formed hydroperoxides (ROMs) to generate in vitro alkoxyl (RO^●^) and peroxyl (ROO^●^) radicals in the presence of iron released from serum by an acidic buffer was measured (d-ROMS test, cod. MC001, DIACRON Labs S.r.l., Grosseto, Italy). Water, calibrator, or serum samples (1 μL) were added into the wells, followed by 2 μL of the chromogenic mixture (aromatic alkyl-amine) and 200 μL of acetate buffer pH 4.8. After careful mixing, the plate was incubated at 37 °C for 90 min and the optical densities were read at λ = 540 nm in an automatic microplate reader. The lyophilized calibrator was stated to contain 320 UCarr. As 1 UCarr is equivalent to 0.08 mg/dL H_2_O_2_, the calibrator concentration was calculated to be 256 mg/L H_2_O_2_, that is, 7.53 mM. The results of the d-ROMS test were expressed according to the following formula:

[Sample] mM H_2_O_2_ = [Abs sample/Abs calibrator] × [calibrator ] (mM H_2_O_2_)

The sensitivity of the d-ROMS test was 0.26 mM H_2_O_2_, and the method was linear up to 267 mM. Intra-andinterassay CV’s were <5%.

### 4.6. Peripheral Blood Lymphocyte Preparation

Isolated lymphocytes, obtained with the above described procedure, were washed twice in phosphate buffered saline solution (0.1 mol/L; pH 7.4); an aliquot of cells was left to dry on a glass slide at room temperature and then were either used for May Grünwald-Giemsa staining, following standard protocol, or for immunofluorescence studies.

### 4.7. Immunofluorescence Analyses

For immunofluorescence studies, the slides were first fixed in cold acetone for 10 min, and subsequently washed in tris buffered saline (TBS) 1×. The lymphocytes were incubated in 1% bovine serum albumin (BSA) for 1 h at room temperature and then overnight at 4 °C with primary antibodies against rabbit polyclonal NOS2 (iNOS) (diluted 1:50; Santa Cruz Biotechnology, Inc., Dallas, TX, USA) or mouse monoclonal cytochrome C (diluted 1:200; Santa Cruz Biotechnology, Inc., Dallas, TX, USA). Thereafter, antibody detection was carried out with secondary antibodies using, respectively, anti-rabbit Alexa Fluor 488 or anti-mouse Alexa Fluor 546 conjugated secondary antibodies (1:200, Invitrogen, UK). Finally, the lymphocytes were counter-stained with DAPI, mounted, and observed with a confocal microscope (510 Meta Zeiss, Germany) as previously described by Rodella et al. [38]. To confirm the specificity of the above stated antibodies, some slides were examined after omitting the primary antibody and in the presence of isotype matched total IgGs, and were then processed as described earlier.

Immunopositivity (staining intensity) of both immunofluorescence analyses were evaluated by measuring 25 random lymphocytes for each patient at each time of blood withdrawal. The quantitative analyses were computed by two independent observers blinded to the patient treatment using an optical fluorescent microscope equipped with an image analyser (Image Pro Plus, Milan, Italy).

### 4.8. Statistical Analysis

Sample size calculations were not done for the present study; all patients enrolled in the MelaSed study [27] presenting a mechanical ventilation length estimated at ICU admission higher than 8 days were invited to participate in this part of the study. Sex was not considered a factor in the statistical analysis of the data.

The patients’ baseline characteristics and single-observation outcomes were analysed by Student’s *t*-test, Wilcoxon rank-sum test, Poisson regression, and by the Fisher exact test, according to the variable type and to the normality of the distribution. Analyses for repeated measures were performed for outcomes recorded repeatedly during the ICU stay. Comparisons were made by population-averaged Poisson models or by cross-sectional time-series regression models for the repeated measures. Statistical analyses were performed with the statistical package Stata 12 (Stata Corporation, College Station, TX, USA).

## 5. Conclusions

Enteral administration of melatonin was adequate to obtain pharmacological levels even in the early phase of critically-illnesses, with a favourable pharmacokinetic profile. The administration of melatonin enhanced the total antioxidant capacity of the blood, with a potential beneficial role in critically-ill patients, due to its immunomodulatory and antioxidant properties.

Since melatonin supplementation could be useful in ICU patients, without clinical side effects [27] and with a decreased oxidative stress proportional to higher blood levels, we suggest an enteral administration of doses adequate to maintain pharmacological values during the whole night period. This goal could be achieved by the administration of 3 mg twice (for example: 20:00 and midnight), or with a dosage not lower than 10 mg in one only evening administration.

## Figures and Tables

**Table 1:** Clinical and demographic characteristics of the studied patients. Statistical tests have been performed on baseline randomized data. Analysis was made by Student *t*-test or Pearson’s chi-squared test. *N*: number of patients; SD: standard deviation; SAPS: Simplified Acute Physiology Score; ICU: Intensive Care Unit; LOS: length of stay.

Characteristic	Placebo (*N* = 29)	Melatonin (*N* = 35)	*p*
Age in years—mean (range)	65 (23 ; 84)	68 (24 ; 83)	0.474
Male sex—*n* (%)	17 (58.6)	19 (54.3)	0.728
SAPS II score at admission—mean (SD)	46.8 (15.1)	44.1 (16.6)	0.399
Admission type—*n* (%)			
Medical	21 (72.4)	24 (68.6)	
Surgical scheduled	3 (10.3)	3 (8.6)	0.847
Surgical unscheduled	5 (17.2)	8 (22.9)	
Diagnosis—*n* (%)			
Pneumonia—Lung diseases	12 (41.4)	16 (45.7)	
Pancreatic diseases	5 (17.2)	6 (17.1)	
Gastrointestinal diseases	4 (13.8)	4 (11.4)	0.998
Acute myocardial infarction—Heart failure	3 (10.3)	3 (8.6)	
Circulatory arrest—Severe arrhythmia	2 (6.9)	3 (8.6)	
Others	3 (10.3)	3 (8.6)	
Septic state—*n* (%)			
None	8 (29.6)	12 (35.3)	
Systemic Inflammatory Response Syndrome	5 (18.5)	5 (14.7)	0.290
Sepsis	3 (11.1)	10 (29.4)	
Severe sepsis	2 (7.4)	2 (5.9)	
Septic shock	9 (33.3)	5 (14.7)	
ICU–LOS in days—mean (SD)	22.8 (18.8)	21.3 (23.6)	0.450
Mechanical ventilation length in days—mean (SD)	20.4 (19.3)	16.6 (21.1)	0.219
ICU mortality—*n* (%)	10 (34.5)	8 (22.9)	0.300

## Supplementary Materials

Supplementary materials can be found at www.mdpi.com/1422-0067/18/4/759/s1.

## Abbreviations

ICU	Intensive Care Unit
SAPS II	Simplified Acute Physiology Score
SIRS	Systemic Inflammatory Response Syndrome
LOS	Length Of Stay
TAC	Total Antioxidant Capacity
iNOS	inducible Nitric Oxidase Synthase
GSH	Glutathione
RPMI	Roswell Park Memorial Institute
DMSO	Dimethylsulphoxide
ELISA	Enzyme Linked ImmunoSorbent Assay
SPE	Solid-Phase Extraction
RP	Reversed Phase
ROMs	Reactive Oxygen Metabolites
TBS	Tris Buffered Saline
BSA	Bovine Serum Albumin
DAPI	4′,6-diamidino-2-phenylindole
