# Supplementary material for: Melatonin Pharmacological Blood Levels Increase Total Antioxidant Capacity in Critically Ill Patients
Source: Int J Mol Sci. 2017 Apr 3;18(4):759. doi: 10.3390/ijms18040759 (PMC5412344; doi:10.3390/ijms18040759)
Supplement: Supplementary file 1 [file ijms-18-00759-s001.docx]

Supplementary Materials: Melatonin Pharmacological Blood Levels Increase Total Antioxidant Capacity in Critically Ill Patients

Giovanni Mistraletti, Rita Paroni, Michele Umbrello, Lara D’Amato, Giovanni Sabbatini, Martina Taverna, Paolo Formenti, Elena Finati, Gaia Favero, Francesca Bonomini, Rita Rezzani, Russel J. Reiter and Gaetano Iapichino


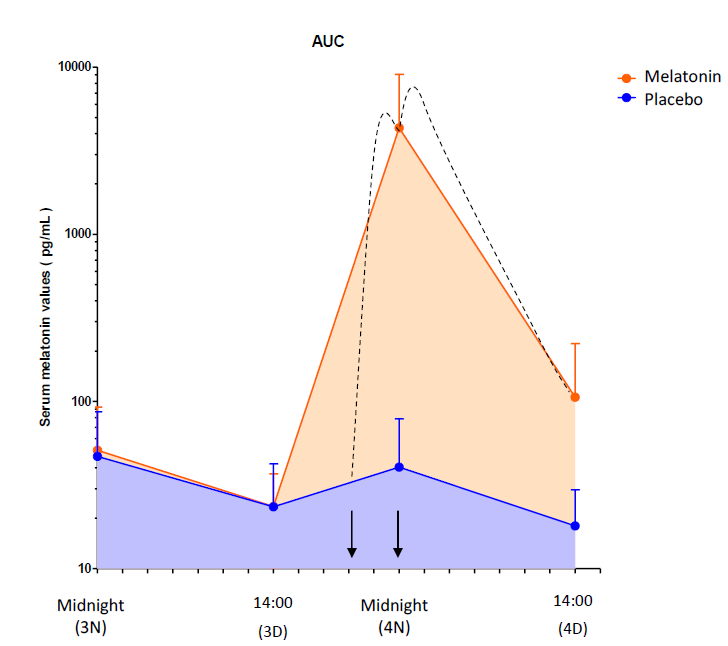


**Figure S1.** Area under the melatonin serum concentration/time curve in the baseline and post-treatment early phase (from midnight of the 3rd ICU day to 14:00 h of the 4th ICU day). The black arrows indicate the first two melatonin or placebo administrations. In the early phase of the study, blood was collected after 4 and 18 h from the first melatonin administration. The AUC of the melatonin treated patients was 53,454 ± 57,103 (mean ± SD, *n* = 21), while for the placebo (*n* = 23) it was 1221 ± 825 pg·h/mL, *p* < 0.001. The dashed line describes the hypothetical pharmacokinetics curve due to the repeated melatonin administrations.

**Figure S2.** Immunopositivity of lymphocyte inducible nitric oxide synthase (iNOS) and cytochrome C (ctox) variations from the 3rd ICU day, measured in critically-ill patients. N denotes night and D denotes day. * denotes *p* < 0.05 between groups.

**Table S1.** Serum melatonin and total antioxidant capacity sampled at different times, in critically-ill patients. Analysis was performed by Wilcoxon rank-sum test for single observations. TAC: total antioxidant capacity; N: night and D: day.

|  | **Sampling Time** | ***n*** | **Group P** | ***n*** | **Group M** | ***p*** |
| --- | --- | --- | --- | --- | --- | --- |
| Melatonin  (pg·mL^−1^) | 3N | 22 | 33.8 [25.0 ; 62.2] | 21 | 32.0 [21.0 ; 57.0] | 0.923 |
|  | 3D | 22 | 16.8 [13.0 ; 23.0] | 21 | 21.0 [13.0 ; 31.6] | 0.743 |
|  | 4N | 22 | 20.3 [14.7 ; 62.3] | 19 | 2514.0 [982.3 ; 7148.0] | <0.001 |
|  | 4D | 22 | 14.3 [11.0 ; 24.4] | 19 | 51.0 [23.0 ; 180.0] | 0.001 |
|  | 8N | 22 | 20 [11.5 ; 34.5] | 21 | 75.0 [333.0 ; 3545.0] | <0.001 |
|  | 8D | 22 | 10.9 [7.0 ; 21.6] | 21 | 31.0 [15.0 ; 51.0] | 0.001 |
| TAC  (mmol·Trolox eq·L^−1^·serum) | 3N | 20 | 28.5 [23.0 ; 59.8] | 18 | 33.1 [22.9 ; 56.6] | 0.650 |
|  | 3D | 20 | 24.9 [21.6 ; 55.6] | 18 | 28.2 [18.0 ; 59.5] | 0.884 |
|  | 4N | 18 | 60.8 [42.4 ; 89.0] | 21 | 106.8 [97.5 ; 123.3] | <0.001 |
|  | 4D | 18 | 27.7 [24.6 ; 50.4] | 17 | 37.3 [30.3 ; 69.3] | 0.092 |
|  | 8N | 19 | 66.9 [51.7 ; 94.9] | 21 | 123.3 [80.5 ; 156.8] | <0.001 |
|  | 8D | 20 | 30.1 [23.8 ; 61.9] | 20 | 35.2 [21.3 ; 72.6] | 0.882 |

**Table S2.** Melatonin and total antioxidant capacity variation from the 3rd ICU day (baseline) to the 4th (early) and 8th (late) ICU days, after the beginning of melatonin or placebo treatment, measured in critically-ill patients. Analysis was performed by Wilcoxon rank-sum test. TAC: total antioxidant capacity; N: night and D: day.

|  | **Delta** | ***n*** | **Placebo** | ***n*** | **Melatonin** | ***p* between Groups** |
| --- | --- | --- | --- | --- | --- | --- |
| Melatonin | 4N | 22 | −3.4 [−22.1 ; 6.9] | 19 | 2475.0 [835.9 ; 7130.0] | <0.001 |
|  | 4D | 22 | −3.4 [−12.7 ; 5.4] | 19 | 34.4 [0.0 ; 147.0] | <0.001 |
|  | *p* within the group |  | 0.592 |  | <0.001 |  |
|  | 8N | 22 | −10.5 [−51.0 ; 3.3] | 21 | 678.9 [319.0 ; 3522.4] | <0.001 |
|  | 8D | 22 | −5.5 [−17.0 ; 1.0] | 21 | 6 [−7.6 ; 36.0] | 0.008 |
|  | *p* within the group |  | 0.148 |  | <0.001 |  |
| TAC | 4N | 16 | 23.6 [2.7 ; 33.5] | 18 | 61.5 [51.3 ; 75.7] | <0.001 |
|  | 4D | 17 | −0.9 [−10.8 ; 7.2] | 15 | 9.8 [1.1 ; 19.7] | 0.062 |
|  | *p* within the group |  | 0.005 |  | 0.002 |  |
|  | 8N | 18 | 25.3 [16.5 ; 42.8] | 18 | 82.9 [47.0 ; 116.1] | <0.001 |
|  | 8D | 20 | 5.3 [−1.3 ; 7.4] | 18 | 7.7 [−1.5 ; 11.2] | 0.447 |
|  | *p* within the group |  | 0.002 |  | <0.001 |  |

**Table S3.** Immunopositivity of lymphocyte inducible nitric oxide synthase and cytochrome C oxidase in critically-ill patients, sampled at different time points. Analysis was performed by Wilcoxon rank-sum test. iNOS: lymphocytes inducible nitric oxide synthase; Cytochrome C: lymphocytes cytochrome C; N: night and D: day.

|  | **Sampling Time** | ***n*** | **Placebo** | ***n*** | **Melatonin** | ***p*** |
| --- | --- | --- | --- | --- | --- | --- |
| iNOS | 3N | 6 | 16.3 [15.3 ; 18.9] | 20 | 15.5 [12.9 ; 17.4] | 0.465 |
|  | 3D | 12 | 16.2 [10.7 ; 18.7] | 21 | 16.9 [15.5 ; 20.1] | 0.477 |
|  | 4N | 18 | 20.9 [15.0 ; 23.8] | 21 | 20.0 [17.0 ; 22.5] | 0.977 |
|  | 4D | 16 | 20.2 [12.7 ; 25.1] | 18 | 13.6 [12.0 ; 20.0] | 0.129 |
|  | 8N | 10 | 18.4 [13.9 ; 21.6] | 15 | 13.8 [11.0 ; 18.9] | 0.059 |
|  | 8D | 10 | 19.3 [13.6 ; 24.1] | 10 | 19.0 [13.9 ; 21.1] | 0.94 |
| Cytochrome C | 3N | 11 | 10.2 [8.5 ; 12.2] | 21 | 11.1 [8.2 ; 15.5] | 0.858 |
|  | 3D | 14 | 5.9 [4.0 ; 8.0] | 21 | 10.7 [6.6 ; 12.3] | 0.005 |
|  | 4N | 17 | 8.4 [6.6 ; 10.0] | 22 | 6.4 [5.2 ; 12.5] | 0.263 |
|  | 4D | 18 | 8.2 [5.5 ; 9.6] | 23 | 7.7 [4.4 ; 13.7] | 0.906 |
|  | 8N | 13 | 3.6 [2.1 ; 7.9] | 11 | 4.4 [1.2 ; 7.5] | 0.977 |
|  | 8D | 13 | 12.5 [6.3 ; 19.3] | 13 | 3.6 [3.0 ; 7.0] | 0.069 |

**Table S4.** Variation in lymphocytes inducible nitric oxide synthase and cytochrome C immunopositivities from the 3rd ICU day (baseline) to the 4th (early) and 8th (late) ICU days, after the beginning of melatonin or placebo treatment, measured in critically-ill patients. Analysis was performed by Wilcoxon rank-sum test. iNOS: inducible nitric oxide synthase; N: night and D: day.

|  | **Delta** | ***n*** | **Placebo** | ***n*** | **Melatonin** | ***p* between Groups** |
| --- | --- | --- | --- | --- | --- | --- |
| iNOS | 4N | 5 | 1.8 [1.2 ; 2.6] | 15 | 5.9 [0.2 ; 11.1] | 0.239 |
|  | 4D | 11 | 3.7 [0.2 ; 8.8] | 15 | −2.9 [−6.5 ; 2.2] | 0.027 |
|  | *p* within the group |  | 0.317 |  | 0.328 |  |
|  | 8N | 1 | 2.7 [2.7 ; 2.7] | 10 | −1.8 [−3.9 ; 4] | 0.527 |
|  | 8D | 8 | 6.1 [1.7 ; 9.2] | 8 | 1.1 [−1.7 ; 6.5] | 0.345 |
|  | *p* within the group |  | 0.317 |  | 0.028 |  |
| Cytochrome C | 4N | 10 | −4.1 [−8.3 ; 1.3] | 17 | −0.6 [−4.1 ; 2.5] | 0.315 |
|  | 4D | 14 | 1.1 [−2.1 ; 5.8] | 17 | 0.1 [−5.1 ; 1.8] | 0.218 |
|  | *p* within the group |  | 0.139 |  | 0.683 |  |
|  | 8N | 9 | −8.3 [−9.5 ; −3.4] | 8 | −2.2 [−6.8 ; −1.0] | 0.386 |
|  | 8D | 11 | 7 [−2.6 ; 14.1] | 9 | −1.1 [−5.1 ; 2.7] | 0.138 |
|  | *p* within the group |  | 0.036 |  | <0.001 |  |
